# The abdominal compliance index and postoperative pain after laparoscopic gynecologic surgery: a preliminary observational cohort study

**DOI:** 10.55730/1300-0144.5969

**Published:** 2025-01-15

**Authors:** Ebru Akgün ŞARER, Mustafa TAHTACI, Umut Cahit ERSOY, Mehtap HONCA

**Affiliations:** 1 Department of Anesthesiology, Bilkent City Hospital, Ankara, Turkiye; 2 Department of Gastroenterology, Bilkent City Hospital, Yıldırım Beyazıt University, Ankara, Turkiye

**Keywords:** Laparoscopy, pneumoperitoneum, compliance, shoulder pain

## Abstract

**Background/aim:**

In gynecology, laparoscopic surgery has the advantages of reduced scarring, less postoperative pain, faster recovery, and lower risk of complications. Despite these advantages, shoulder pain still remains a major concern, affecting up to 90% of patients undergoing gynecologic laparoscopic surgery. Use of the abdominal compliance index (ACI) may offer the benefit of increased space for safer surgery and may prevent the drawback of high insufflation pressures. The aim of this study was to investigate the relationship between shoulder pain, abdominal pain, and analgesic use with the recently advised ACI, a surrogate index of abdominal expansion in patients undergoing gynecologic laparoscopic surgery.

**Materials and methods:**

Thirty-one patients with American Society of Anesthesiologists scores of 1–3 who underwent elective gynecologic laparoscopic surgery were included. The insufflation pressure was set to 12 mmHg. ACI, defined as insufflated intraabdominal volume (L) per body surface area (m^2^), was used to estimate the relationship between abdominal compliance and postoperative pain.

**Results:**

The 31 patients were separated into two groups based on median ACI level (range 1.37–2.73 L/m^2^), with those having values of 2.16 L/m^2^ or lower as group 1 and those with higher values as group 2. Abdominal visual analogue scale (VAS) scores at 30 min postoperative were significantly higher in group 2 than in group 1 (p < 0.001). Shoulder pain VAS scores at 24 h and 36 h postoperative were also significantly higher in group 2 than in group 1 (p = 0.021 and p = 0.002, respectively). The total amount of analgesic infusion and additional analgesic requirements were lower in group 1 than in group 2 (p = 0.049 and p = 0.001, respectively). ACI did not differ with patient demographic characteristics or history of abdominal/laparoscopic surgery, parity, abdominal drainage, and pneumoperitoneum time.

**Conclusion:**

ACI, a surrogate index of abdominal expansion capacity, may be used to guide individualization of insufflation pressures by identifying female patients under risk of higher postoperative pain.

## 1. Introduction

Elevated intraabdominal pressure has gained increased attention in surgical and medical settings over recent decades, including in intensive care [1,2]. A pneumoperitoneum at 12 mmHg is an iatrogenically created situation that leads to short-term abdominal hypertension. Full expansion for every patient has certainly not been certainly achieved at one pressure setting. Abdominal compliance is the measure of abdominal expansion and defined as the change in intraabdominal volume per change in intraabdominal pressure (mL/mmHg), which is unique to each patient [3]. The elasticity of the abdomen and diaphragm are the major determinants [4].

An increase in intraabdominal pressure exceeding the limits of abdominal compliance reduces blood flow and impairs perfusion, resulting in ischemia, hypoxia, and increased oxidative stress [1,5]. Reduced abdominal compliance at higher insufflation pressures indicates that the surrounding tissues and organs are exposed to disproportional stress, without substantial improvement of surgical workspace [4].

Normal abdominal compliance is in the range 250–450 mL/mmHg. The accommodation of the abdominal cavity follows phases of reshaping, stretching, and pressurization [5]. The stretching capacity of abdominal expansion is influenced by numerous factors such as age, obesity, gender, fat distribution (visceral vs. subcutaneous), history of abdominal surgery, and chronic medical problems like ascites, peritoneal dialysis, and severe chronic obstructive pulmonary disease. In female patients, parity also plays a role [6–8]. Chronic increased intraabdominal pressure may damage the viscoelastic components of the abdominal wall, thereby increasing abdominal compliance.

The pneumoperitoneum of laparoscopic surgery is determined by biomechanical rules of compliance and pressure as well as physical principles like Pascal’s and Laplace’s laws [9]. The gas pressure settings for a laparoscopic pneumoperitoneum only give rough estimations for abdominal compliance [6,7]. Currently, a simple and practical method for measuring and continuously monitoring abdominal compliance during laparoscopic surgery is not available [6]. Endoscopic oscillometry permits concurrent monitoring of the changes in abdominal compliance throughout laparoscopic surgery in an animal model. It has been validated in opposition to compliance arising from computed tomographic (CT) imaging. The abdominal compliance was calculated from the subsequent pressure and flow in the insufflation circuit by using small oscillations [10].

Selecting the lowest possible intraabdominal pressure is recommended to avoid the probable adverse effects of insufflation pressure on circulation [6]. Nevertheless, in clinical practice, a practical methodology to achieve this has not yet been defined. Diaz-Cambronero et al. suggested a personalized pressure approach where the abdomen is prestretched in 15 mmHg and then the pressure is progressively decreased to the minimum intraabdominal pressure that still preserves optimal surgical workspace [11].

Postoperative pain following laparoscopic surgery comprises incisional trocar pain and pneumoperitoneum pain [6]. Abdominal wall expansion due to the insufflation and diaphragmatic irritation from the acidity of the carbon gas are the contributing factors to pneumoperitoneum pain [6]. Postoperative shoulder pain is one of the most frequent and troublesome problems, with an incidence rate of 34%–82% after laparoscopic gynecologic surgery [12].

The cause of this shoulder pain has not been fully elucidated and is thought to be multifactorial, likely referred pain. The central and diaphragmatic pleura are innervated by the phrenic nerve (C3–C5), while the acromion process is innervated by the supraclavicular nerve (C3–C4). When the phrenic nerve is irritated, pain appears in the neck or scapula [13]. One theory suggests this pain in caused by irritation of the peritoneal and diaphragmatic nerves by carbonic acid, which reduces peritoneal pH [14]. Another theory posits that retained gas and delayed absorption of CO_2_ leads to strain on the triangular and coronary ligaments of the liver, causing subdiaphragmatic pain when patients sit or mobilize, usually more than four hours after the operation [15]. The neuropraxia theory suggests that stretching and/or peritoneal and diaphragmatic injury leads to tearing of blood vessels, strain on the phrenic nerve, and the formation of inflammatory mediators, which evoke referred pain in the shoulder [14].

More recently, a surrogate clinical index for abdominal compliance has been proposed as a feasible predictor of postoperative pain in male patients undergoing laparoscopic inguinal hernia repair [6]. It was verified that the abdominal compliance index (ACI) could be obtained from precisely calculated abdominal compliance with high predictive correctness. We speculate that in patients with higher ACI values, greater expansion of the abdomen leads to extreme stretching of the abdominal wall and causes the patient to experience more severe pain. The ACI has probable clinical suggestions for adjusting intraabdominal pressure. It could be a useful surrogate clinical index to predict optimal pressure settings while reducing surgical stress in association with pneumoperitoneum. We aim to investigate a newly defined surrogate index of abdominal compliance in relation to postoperative abdominal and shoulder pain in patients undergoing elective laparoscopic gynecologic surgery.

## 2. Materials and methods

This study was approved by the Ethics Committee of Bilkent City Hospital, Ankara, Türkiye with decision number (TABED1-24-321) dated 06/06/2024. Informed consent was acquired from all participating patients to use their clinical data and personal information upon admission.

### 2.1. Study design

The study was a prospective observational cohort study including patients over 18 years of age with an American Society of Anesthesiologists (ASA) score of 1–3 undergoing elective laparoscopic gynecologic surgery. Exclusion criteria included conversion to open surgery; having a body mass index (BMI) >35 kg/m^2^; loss of data during follow-up, chronic pain syndromes like fibromyalgia, neck, or shoulder pain; a history of opioid use; allergies to the study drugs; cognitive impairment; unstable cardiovascular disease; severe chronic obstructive pulmonary disease; liver or kidney dysfunction; or inability to communicate.

### 2.2. Data collection

Postoperative pain was evaluated using a VAS of 0–10 recorded at 30 min, 6 h, 24 h, and 36 h postoperatively. Postoperative pain was classified as abdominal or shoulder pain. Abdominal pain was defined as away from the trocar site and was regarded as visceral (nonincisional) pain. Postoperative analgesic consumption was also documented at the same time intervals.

### 2.3. Anesthesia protocol and surgical procedure

Similar general anesthesia and surgical protocols were applied to all patients. None of the patients were premedicated. Heart rate, noninvasive blood pressure, and oxygen saturation were monitored upon entrance to the operation room. After preoxygenation, general anesthesia was induced with propofol (2.5 mg/kg) and fentanyl (2 μg/kg). Tracheal intubation and muscle relaxation was facilitated by rocuronium (0.9 mg/kg) and repeated as necessary at periodic intervals. Anesthesia was maintained with 1 MAC desflurane with oxygen in the air (1:2 ratio) and remifentanil infusion (0–1–0.5 μg/kg/min). All patients were ventilated to maintain end tidal CO_2_ at 35–40 mmHg. Respiratory rate and tidal volume were adjusted accordingly. Bispectral index levels were kept at 40–60. Ondansetron (8 mg IV) was administered for nausea and vomiting prophylaxis when the surgeons started to close the umbilical trocar sites. Neuromuscular blockade was reversed with sugammadex (2 mg/kg). Patients were set in the steep Trendelenburg and lithotomy positions during surgery.

All surgeries were performed by experienced laparoscopic surgeons with abdominal insufflation at 12 mmHg using a standard automatic insufflator unit with a flow rate of 3 L/min (Karl Storz, Germany). The insufflated intraabdominal volume (IAV) was measured from the cumulative CO_2_ volume gauge on the insufflation module of the laparoscopy system. A standard technique was used with one 10-mm trocar and two 5-mm trocars. The insufflated gas was not heated or humidified with additional devices. The initial insufflation volume was used to measure abdominal compliance after equilibrium and divided by body surface area (BSA) using the Du Bois formula to adjust for body constitution. The resulting surrogate index represented the abdominal expansion capacity per unit area under 12 mmHg insufflation pressure [16]. The ACI was calculated as follows:


$$\text{ACI }(\text{L}/{\text{m}}^{2})=\text{insufflated IAV }(\text{L})/(0.007184\times {\text{height}}^{0.725}\;(\text{cm})\times {\text{weight}}^{0.425}\;(\text{kg}))$$

For all patients, the remainder of the surgery was performed with intraabdominal gas pressure held at 12 mmHg, and residual carbon dioxide was completely evacuated at the end of the procedure by manual squeezing of the abdomen with open trocars. Patients were monitored in the postoperative care unit (PACU) until their conditions stabilized. Patient demographics, operation duration, and pneumoperitoneum time were recorded.

### 2.4. Postoperative analgesia

The use of a patient-controlled analgesia (PCA) device and the VAS for postoperative pain assessment were explained to all patients during the preoperative visit. Abdominal pain and shoulder pain were questioned separately at 30 min after surgery and then at predefined time intervals (6, 12, 24, and 36 h postoperatively) using a 10-cm VAS scaled from 0 = no pain to 10 = unbearable pain. The standard analgesic protocol of tramadol (2 mg/kg) and paracetamol (1 g IV) was applied approximately 20 min before the end of the surgery. Tilcotil (20 mg IV) was administered upon request. For all patients, a PCA device was started in the PACU to deliver a bolus dose of 10 mg tramadol with a 15-min lock interval without a basal infusion. Ondansetron (IV) was given every 8 h for 24 h postoperatively. For VAS scores >3, patients were treated with rescue analgesics: paracetamol (1g IV) or tilcotil (20 mg IV). Tramadol requests, cumulative amounts, and total tramadol requirements were recorded from PCA memory and additional analgesic uses were recorded at the specified time intervals.

### 2.5. Statistical analysis

A power analysis was conducted using G*Power v.3.1.9.7 (Franz Faul, Universitat Kiel, Germany), resulting in n1 = 15 (3.2 ± 0.4), n2 = 16 (4.3 ± 0.4), standard deviation = 0.7, α = 0.05, and effect size (d) = 1.57. The power was found to be 98%.

Preliminary analyses were completed for frequencies, means, standard errors (SE), and percentages as applicable. A histogram and the Shapiro–Wilk test were used to analyze the variable distributions. Categorical variables were analyzed using the chi-square test, and continuous variables were analyzed using the Mann–Whitney U test. Statistical significance was set at p < 0.05 for all analyses. The statistical analyses were done using SPSS v.17.0 (SPSS Inc.; Chicago, IL, USA).

## 3. Results

A total of 31 patients participated in the study. Three patients were excluded, two due to conversion to open surgery and one due to surgery cancellation. The patient and procedural demographics are shown in Table 1. The age range of the patients was 27–78, with a mean age of 53.03 ± 11.9 years. The patients were grouped by median ACI level, which ranged from 1.37 to 2.73 L/m^2^. Group 1 included those with an ACI ≤ 2.16 L/m^2^, and group 2 included those with an ACI > 2.16 L/m^2^. There were no significant differences in age, height, weight, BMI, BSA, surgery history, comorbidities, delivery history, mode of delivery, abdominal drainage placement, ASA score, or pneumoperitoneum time between the two groups (p > 0.05).

**Figure 1:**
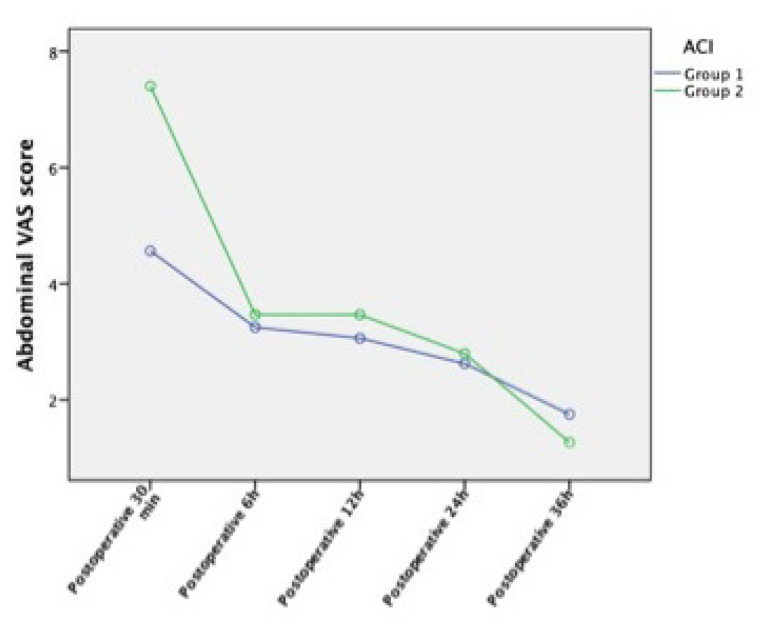
Comparison of abdominal VAS scores at different postoperative time points. ACI = abdominal compliance index and VAS = visual analogue scale.

**Figure 2:**
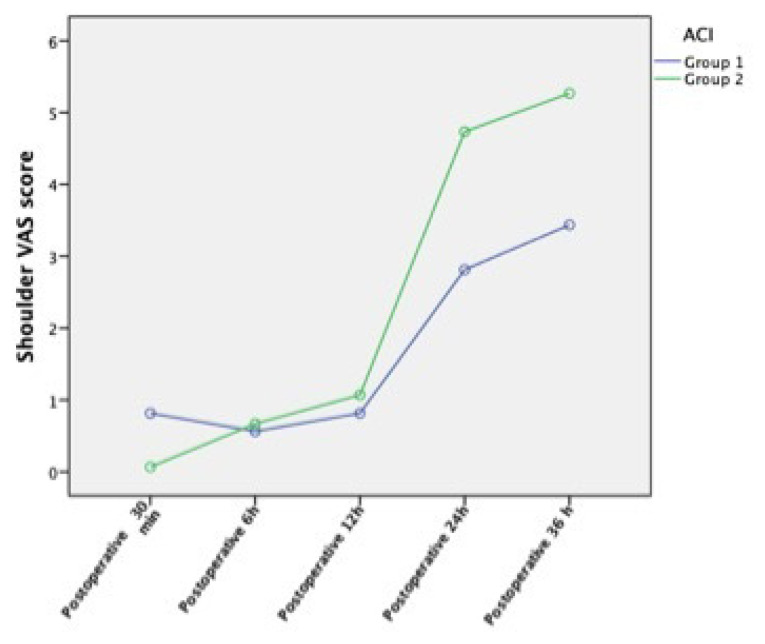
Comparison of shoulder VAS scores at different postoperative time points. ACI = abdominal compliance index and VAS = visual analogue scale.

The total amount of tramadol infusion and the insufflated volume (TV) were significantly lower in group 1 than in group 2 (p = 0.049 and p < 0.001, respectively) (Table 2). Additional analgesic use was higher in group 2 than group 1 (p = 0.001). Abdominal pain VAS scores at 30 m postoperative were significantly higher in group 2 than in group 1 (p < 0.001). Shoulder pain VAS scores at 24 h and 36 h postoperative were significantly higher in group 2 than in group 1 (p = 0.021 and p = 0.002, respectively). The number of patients experiencing abdominal and shoulder pain with VAS scores over 3 at different time points is shown in Table 3. A comparison of abdominal VAS scores at different time points is presented in Figure 1, and a similar comparison for shoulder pain VAS scores is presented in Figure 2.

## 4. Discussion

The impact of pneumoperitoneum pressure on postoperative pain is broadly emphasized, but the relationship between pain after pneumoperitoneum and abdominal compliance due to expansion has thus far not been evaluated in laparoscopic gynecologic surgery.

Abdominal compliance is a largely overlooked factor in intensive care, robotic surgery, and laparoscopic surgery [5]. The relationship between intraabdominal pressure with IAV is not linear as the pressure-volume curve is nonlinear. Its slope shows the facility of abdominal expansion defined as abdominal compliance [17]. Until complete expansion is reached, more gas means more space. After maximal compliance is attained, more gas increases pressure without any improvement in operating workspace and influences end organ perfusion for a limited time, creating short term abdominal hypertension [7,18]. One pressure setting does not fit all patients. In other words, the amount of gas needed to establish the optimum surgical workspace is specific for any given patient.

As a first in the literature, the findings of this study demonstrate that high ACI is significantly related to postoperative abdominal pain at 30 min and to postoperative shoulder pain at 24 h and 36 h after laparoscopic gynecologic surgery. Postoperative analgesic consumption and additional analgesic requirements were also increased in the high ACI group, meaning patients with greater abdominal expansion at 12 mmHg intraabdominal pressure.

The orientation of connective tissue fibers, the biomechanics of the abdominal wall, and physical principles combine to form abdominal compliance. It is an anisotropic, nonlinear, and dynamic entity [19]. Insufflation of the abdominal wall during pneumoperitoneum switches the potential intraabdominal space from a flattened sphere to an elliptical one. It has overlapping phases of reshaping, stretching, and pressurization. The abdominal cavity, which is a dome-shaped container, acts as a hydraulic system [18,20]. Normal abdominal pressure at rest in a supine position is 5–7 mmHg. Stretching of the anterolateral abdominal muscles increases the anteroposterior diameter of the abdomen and decreases the transverse diameter. After the maximal stretch and elastic expansion of the abdominal wall is reached, small increments in volume leads to dramatic increments in intraabdominal pressure in the pressurization phase [7,21,22].

The disadvantages of CO_2_ insufflation might be attenuated by avoiding the use of extreme pressures through monitoring abdominal compliance. Lower pneumoperitoneum pressure is related to lower postoperative pain [23–26]. The lowest intraabdominal pressure under maximal abdominal wall compliance creates ideal circumstances for laparoscopic surgery by minimizing the pathophysiological effects of excessive pressurization while providing sufficient surgical exposure [7]. Accurate calculation of abdominal compliance requires multiple measurement sites with special instruments and equipment such as flowmeters, tensiometers, and computer recording systems, making it unfeasible in a clinical setting [6].

There is no clinically available practical method to measure and guide the tension/stress balance exerted on the intraabdominal organs and tissues [10]. Calculating the insufflated CO_2_ gas volume has disadvantages, such as gas leakage through trocars, removal through suction, and the absorption of gas by the peritoneum over time. Magnetic resonance imaging and CT have been used experimentally to obtain precise and repeatable calculations, but their added benefit is disclaimed by their drawbacks. Interference with the surgery, additional time required for measurements, and the presence of ionizing radiation or powerful magnetic fields prevent their routine use in clinical settings. Endoscopic oscillometry, a new strategy offering quantitative analysis of abdominal compliance alterations through stepwise insufflation, may also not be clinically practical since it requires preparations for endoscopic measurements [10].

Females perceive postoperative pain more severely than males. Parity and history of past laparoscopic and abdominal surgery affects the intensity of postoperative pain [8,24,27,28]. Kinoshita et al. recently noted that novel ACI might not correctly predict postoperative pain in patients with a history of laparoscopic surgery or pregnancy since intraabdominal capacity increases and abdominal elasticity decreases [6]. Actual ACI is decreased, while ACI would presumably increase [4]. No differences were found between the groups in this study in terms of parity and history of abdominal and laparoscopic surgery.

Jiang et al. found a negative correlation between pain scores and BMI, with BMI values <24 being an independent risk factor for shoulder pain after laparoscopic surgery [12]. One possible explanation for this finding is that the stretching of the CO_2_ gas irritating the diaphragm fills a larger space in the upper abdomen of thin patients than those of obese patients, whose upper abdominal area is covered by omentum [13]. Patients with BMI values >35 kg/m^2^ were not included in this study. BMI neither affected the results nor differed between the groups.

Kim et al. emphasized that the risk of postoperative shoulder pain increases with decreased height, weight, and BMI and with greater abdominal circumference difference in a univariate analysis of patients after laparoscopic appendectomy [29]. High ACI patients, who have larger IAV per unit body area, may have increased residual gas pockets in the abdominal cavity. More abdominal expansion might result in more tensile stress, causing higher postoperative pain in these patients. There is a positive correlation between the intensity of shoulder pain and volume of residual gas [30]. No direct measurement of the residual gas was done in the current study.

The abdominal pain VAS score at 30 min postoperative is significantly higher in group 2. This might be due to excessive stretching of the pneumoperitoneum and more retained gas in the abdomen. Several studies have emphasized that time characteristics of postoperative abdominal and shoulder pain differ after laparoscopic surgery. Abdominal pain peaks in the first 24 h postoperative and then decreases, while shoulder pain that is minimal on the day of surgery increases the following day [31]. This study followed that same pattern, with postoperative shoulder pain worsening over time. Li et al. found that shoulder pain is the most resistant type of pain to nonsteroidal anti-inflammatory drugs and opioid analgesics [32], which is consistent with our findings. Despite the use of additional analgesics, shoulder pain increased over time in our patients. Analgesic administration protocols should be organized according to the characteristics of shoulder pain occurrence [31,32].

The precise mechanism of shoulder pain after laparoscopic surgery has not been elucidated. A main explanation for referred pain to the shoulder mediated by the phrenic nerve is the extreme stretching of the diaphragm caused by the pressure of the insufflated gas to establish pneumoperitoneum. Subcostal residual carbonic gas is also thought to be responsible, irritating phrenic nerves with its acidic nature [6].

A current review emphasized several methods for reducing or preventing the severity of shoulder pain. These strategies embrace the use of alternative insufflating gases to establish pneumoperitoneum, the use of warmed and humidified gas, the use of drugs preventatively or therapeutically, intraperitoneal application of local anesthetic agents, the use of intraperitoneal drains, and the instillation of intraperitoneal fluid. For the ejection of gas from the abdominal cavity, techniques such as the pulmonary recruitment maneuver, a postoperative Trendelenburg position, or active suction and manual evacuation of gas have been used [14,33–39]. Identifying patients most likely develop shoulder pain after laparoscopy may allow for sustainable and individualized strategies to minimize pain. Unfortunately, the literature has shown diverse and occasionally contradictory results concerning the efficacy of these interventions.

One limitation of this study is that we used the Du Bois BSA formula, which is calculated using only height and weight. Thus, adipose tissue and muscle mass could not be differentiated. Other limitations were that the study population was composed of patients with both benign and malign gynecological diseases, and there was a small sample size.

This study is the first preliminary prospective cohort study to show a high association of ACI with postoperative abdominal pain, shoulder pain, and postoperative analgesic consumption after laparoscopic gynecological surgery. ACI, a surrogate index of abdominal expansion capacity, may be used to guide individualization of insufflation pressures by identifying female patients at risk of higher postoperative pain.

## Figures and Tables

**Table 1:** Comparison of patient demographic and procedural characteristics.

	ACI ≤ 2.16 (n = 16)	ACI > 2.16 (n = 15)	p

Age (years)	55.8 ± 3.09	50 ± 2.89	0.202

Height (cm)	161.7 ± 1.27	161.6 ± 1.15	0.861

Weight (kg)	73 ± 2.27	70.2 ± 3.21	0.446

BMI (kg/m^2^)	27.9 ± 0.99	26.7 ± 1.03	0.358

BSA (m^2^)	1.77 ± 0.02	1.74 ± 0.03	0.572

Abdominal/laparoscopic surgical history	8 (50)	5 (33.3)	0.172

Comorbidity	11 (68.8)	14 (93.3)	0.565

Delivery history	13 (81.3)	14 (93.3)	0.6

Delivery mode:			0.143
Vaginal	11 (68.8)	7 (46.7)	
Cesarean	1 (6.3)	6 (40)	
Both	1 (6.3)	1 (6.7)	

ASA score:			0.809
2	10 (62.5)	10 (66.7)	
3	6 (37.5)	5 (33.3)	

Abdominal drain placement	9 (56.3)	7 (46.7)	0.862

Procedure duration (min)	267.62 ± 37.04	196.4 ± 21.24	0.163

Pneumoperitoneum time (min)	223.06 ± 34.29	140.93 ± 22.11	0.086

Values are expressed as n (%) or mean ± SE. ACI = abdominal compliance index, BMI = body mass index, BSA = body surface area, and ASA = American Society of Anesthesiologists.

**Table 2:** Comparison of patient pain characteristics.

	ACI ≤ 2.16 (n = 16)	ACI > 2.16 (n = 15)	p
Abdominal VAS score postoperative 30 min	4.56 ± 0.25	7.40 ± 0.3	<0.001
Abdominal VAS score postoperative 6 h	3.25 ± 0.39	3.46 ± 0.33	0.892
Abdominal VAS score postoperative 12 h	3.06 ± 0.38	3.46 ± 0.54	0.711
Abdominal VAS score postoperative 24 h	2.62 ± 0.23	2.8 ± 0.48	0.711
Abdominal VAS score postoperative 48 h	1.75 ± 0.28	1.28 ± 0.26	0.216
Shoulder VAS score postoperative 30 min	0.81 ± 0.33	0.06 ± 0.06	0.216
Shoulder VAS score postoperative 6 h	0.56 ± 0.32	0.66 ± 0.45	0.682
Shoulder VAS score postoperative 12 h	0.81 ± 0.37	1.06 ± 0.51	0.892
Shoulder VAS score postoperative 24 h	2.81 ± 0.5	4.73 ± 0.67	0.021
Shoulder VAS score postoperative 36 h	3.43 ± 0.51	5.26 ± 0.45	0.002
Total amount of analgesic infusion (mg)	131.2 ± 17.56	183.01 ± 18.37	0.049
Additional analgesic use	1.68 ± 0.21	3.46 ± 0.33	0.001
TV (L)	3.25 ± 0.11	4.35 ± 0.10	<0.001

Values are expressed as mean ± SE. ACI = abdominal compliance index, VAS = visual analogue scale, and TV = insufflated volume.

**Table 3:** Patients with VAS scores > 3 for abdominal and shoulder pain.

	ACI ≤ 2.16 (n = 16)	ACI > 2.16 (n = 15)	p
Abdominal pain postoperative 30 min	16 (100)	15 (100)	NA
Abdominal pain postoperative 6 h	11 (68.8)	13 (86.7)	0.394
Abdominal pain postoperative 12 h	11 (68.8)	10 (66.7)	0.901
Abdominal pain postoperative 24 h	9 (56.3)	7 (46.7)	0.862
Abdominal pain postoperative 36 h	4 (25)	1 (6.7)	0.333
Shoulder pain postoperative 30 min	2 (12.5)	0 (0)	0.484
Shoulder pain postoperative 6 h	1 (6.3)	2 (13.3)	0.600
Shoulder pain postoperative 12 h	3 (18.8)	3 (20)	0.930
Shoulder pain postoperative 24 h	9 (56.3)	13 (86.7)	0.113
Shoulder pain postoperative 36 h	13 (81.3)	14 (93.3)	0.600
